# Responses to increased rates of caesarean births

**DOI:** 10.2471/BLT.25.293141

**Published:** 2025-10-13

**Authors:** Rana Islamiah Zahroh, Meghan A Bohren, Ana Pilar Betrán

**Affiliations:** a Nossal Institute for Global Health, School of Population and Global Health, University of Melbourne, 32 Lincoln Square North, Carlton, Victoria, 3053, Australia.; b UNDP-UNFPA-UNICEF-WHO-World Bank Special Programme of Research, Development and Research Training in Human Reproduction (HRP), World Health Organization, Geneva, Switzerland.

## Abstract

Performing caesarean sections without medical need exposes women and babies to unnecessary risks without clear benefits. Yet the global number of caesarean sections has continued to rise considerably over the years, with caesarean sections increasingly performed before the onset of labour and among women at low risk of birth complications. In recent years, considerable efforts have been made to reduce unnecessary caesarean sections. However, interventions that aim to reduce such births are complex, have mixed outcomes, do not translate well between settings and lack clear evidence on which components or mechanisms drive success. In this article, we outline a three-step pathway for implementing interventions that aim to optimize caesarean use: (i) conduct formative research to identify context-specific needs and priorities; (ii) design evidence-based, multifaceted interventions; and (iii) ensure implementation through meaningful stakeholder engagement. Finally, we emphasize how improving the quality of care during childbirth is key to achieving optimal and equitable use of caesarean sections.

## Résumé

Pratiquer des césariennes sans justification médicale expose les femmes et les bébés à des risques inutiles sans apporter d’avantages évidents. Pour autant, le nombre mondial de césariennes a connu une croissance considérable au fil des ans, celles-ci étant de plus en plus souvent pratiquées avant le début du travail ainsi que chez des femmes présentant un faible risque de complications à l’accouchement. Ces dernières années, des efforts considérables ont été déployés pour réduire le nombre de césariennes inutiles. Toutefois, les interventions qui visent à réduire ces naissances sont complexes, produisent des résultats mitigés, sont difficilement transposables d’un pays à l’autre et ne reposent pas sur des preuves évidentes quant aux éléments ou mécanismes qui déterminent leur réussite. Cet article présente un processus en trois étapes pour la mise en œuvre d’interventions visant à optimiser le recours aux césariennes: (i) mener des recherches formatives afin d’identifier les besoins et priorités spécifiques au contexte; (ii) concevoir des interventions multidimensionnelles fondées sur des données scientifiques; et (iii) garantir la mise en œuvre grâce à une participation importante des parties prenantes. Enfin, nous soulignons à quel point l’amélioration de la qualité des soins pendant l’accouchement est essentielle pour parvenir à un recours optimal et équitable aux césariennes.

## Resumen

Realizar cesáreas sin necesidad médica expone a las mujeres y a los recién nacidos a riesgos innecesarios sin beneficios claros. Sin embargo, el número de cesáreas a nivel mundial ha seguido aumentando considerablemente con el paso de los años, y cada vez se realizan más antes del inicio del trabajo de parto y entre mujeres con bajo riesgo de complicaciones obstétricas. En los últimos años, se han hecho esfuerzos significativos para reducir las cesáreas innecesarias. No obstante, las intervenciones orientadas a disminuir este tipo de partos son complejas, presentan resultados variables, no se adaptan fácilmente a distintos contextos y carecen de evidencia clara sobre los componentes o mecanismos que determinan su éxito. En este artículo, se propone una ruta de tres pasos para implementar intervenciones destinadas a optimizar el uso de la cesárea: (i) realizar investigaciones formativas para identificar las necesidades y prioridades específicas de cada contexto; (ii) diseñar intervenciones multifacéticas basadas en la evidencia; y (iii) garantizar la implementación mediante la participación significativa de las partes interesadas. Finalmente, se destaca que mejorar la calidad de la atención durante el parto es fundamental para lograr un uso óptimo y equitativo de las cesáreas.

## ملخص

إن إجراء عمليات الولادة القيصرية دون ضرورة، يؤدي لتعريض السيدات والأطفال لمخاطر غير ضرورية دون فوائد واضحة. ومع ذلك، استمر العدد العالمي لعمليات الولادة القيصرية في الارتفاع بشكل ملحوظ على مر السنين، مع تزايد إجراء عمليات الولادة القيصرية قبل بدء المخاض، وبين السيدات ذوات الخطر المحدود لمضاعفات الولادة. في السنوات الأخيرة، تم بذل جهود كبيرة للحد من عمليات الولادة القيصرية غير الضرورية. ومع ذلك، فإن التدخلات التي تهدف إلى الحد من هذه الولادات معقدة، ونتائجها متباينة، ولا تُترجم بشكل جيد بين البيئات، وتفتقر إلى أدلة واضحة على المكونات أو الآليات التي تُسهم في النجاح. في هذه المقالة، نقوم بتحديد مسارًا من ثلاث خطوات لتنفيذ التدخلات التي تهدف إلى تحسين اللجوء إلى عمليات الولادة القيصرية: (1) إجراء أبحاث تكوينية لتحديد الاحتياجات والأولويات الخاصة بكل وضع؛ و(2) تصميم تدخلات متعددة الجوانب قائمة على الأدلة؛ و(3) ضمان التنفيذ من خلال اشتراك هادف من أصحاب المصلحة. وأخيرًا، فإننا نؤكد على أهمية تحسين جودة الرعاية أثناء الولادة لتحقيق الاستخدام الأمثل والعادل لعمليات الولادة القيصرية

## 摘要

在没有医疗需求的情况下进行剖腹产，会导致产妇及其胎儿面临不必要的风险，且并无明显益处。然而，全球范围内的剖腹产数量多年以来持续大幅增长，其中在分娩发作之前以及针对分娩并发症风险较低的孕妇进行剖腹产的比例日益上升。近年来，各国政府竭尽全力减少不必要的剖腹产。但是，旨在降低剖腹产率的干预措施非常复杂，效果参差不齐，无法在不同的环境中予以有效实施，且缺乏明确的证据证明哪些干预措施或机制能成功降低剖腹产率。在本文中，我们概述了关于旨在优化剖腹产使用的干预措施的三步走实施途径。(i) 开展形成性研究以确定具体情况下的需求和优先考虑因素；(ii) 制定多方面的循证干预措施；以及 (iii) 通过利益攸关方的有效参与确保干预措施得以实施。最后，我们要强调的是，如何提升分娩期间的护理质量才是实现剖腹产优化和公平使用的关键所在。

## Резюме

Кесарево сечение, проводимое без медицинских показаний, подвергает женщин и детей неоправданным рискам без видимой пользы. Однако во всем мире количество кесаревых сечений неуклонно и в значительной степени растет с течением времени, при этом кесаревы сечения все чаще проводят до начала родовой деятельности и среди женщин с низким риском осложнений при родах. В последние годы стали предприниматься значительные усилия по сокращению числа ненужных кесаревых сечений. Однако вмешательства с целью сокращения количества таких родов сложны, их результаты неоднозначные, они не воспроизводятся в различных условиях, и отсутствует четкая доказательная база в пользу успеха тех или иных компонентов или механизмов. Авторы статьи очерчивают трехэтапный путь внедрения вмешательств, которые имеют целью оптимизацию применения кесарева сечения: (і) проведение подготовительных исследований для выявления контекстно обусловленных потребностей и приоритетов; (іі) разработка основанных на доказательствах многофакторных вмешательств и (ііі) обеспечение внедрения посредством осознанного вовлечения заинтересованных сторон. В заключение авторы подчеркивают, что улучшение качества медицинского ухода при родах является ключевым фактором достижения оптимального и сбалансированного использования кесаревых сечений.

## Introduction

Caesarean births performed without clear clinical need expose women and babies to unnecessary risks and long-term morbidity and mortality, with no evidence of benefit.^1^^,^^2^ Yet caesarean sections are increasingly performed when there is no medical need, particularly before labour begins and among women at low risk of birth complications.^1^^,^^3^ Caesarean sections are also resource-intensive, which means that in low-resource settings they result in a significant burden on the health-care system and may divert resources away from quality care for other important health services.^4^^–^^6^

Although the World Health Organization (WHO) does not promote an ideal caesarean section rate, to reduce avoidable risks to women and babies it emphasizes that caesarean sections should only be performed when medically necessary.^7^^,^^8^ Despite this, the global rate of caesarean births has increased over recent decades, rising from 6% in 1990 to 21% in 2018, and with rates in some countries exceeding 50%.^4^ Researchers predict this trend will continue, reaching over 28% globally in 2030 if no effective interventions are implemented.^4^

Research shows the rise in caesarean sections is driven by interrelated non-clinical factors, involving women, health workers and health systems.^9^ For example, women's fear of labour or desire for auspicious birth dates, combined with health workers’ fear of litigation and financial incentives, can shape preferences for caesarean sections.^9^ This confluence of psychological, professional and economic motivations underscores the complex way in which non-clinical drivers increase caesarean use. The growing contribution of these drivers to the increase in caesarean sections led WHO to develop global guidance on non-clinical interventions to reduce unnecessary caesarean sections.^10^^,^^11^ These interventions (such as childbirth training workshops) occur independently of clinical encounters between health workers and women, and may target women, health workers and/or the broader health system.^10^

Despite WHO’s guidance, real-world implementation of interventions aimed at optimizing caesarean use remains a challenge. Non-clinical interventions can be complex, multicomponent and need multiple actors to change behaviour.^10^^,^^12^^,^^13^ Additionally, there is limited evidence of effectiveness in reducing unnecessary caesarean sections, and interventions do not translate between settings,^10^ likely due to the contextual factors that influence implementation.^14^^,^^15^ Importantly, which specific components of an intervention contribute to its success or failure is often unclear.^14^^,^^15^ In this article, we synthesize existing literature plus findings from recent studies,^10^^,^^11^^,^^14^^–^^16^ and aim to support WHO recommendations by proposing a practical, evidence-informed, three-step pathway to improve the design and implementation of interventions that aim to optimize caesarean use. The pathway can be adapted to local settings and can be used in both public and private health-care sectors; this is particularly important given the private sector’s role in the rising number of caesarean sections in many low- and middle-income countries.

## Step 1: formative research

An important first step when designing interventions for any setting is to understand context-specific factors and determine which populations should be targeted with which interventions. This can be done through formative research. WHO funded the development of a generic protocol for formative research aimed at optimizing caesarean use, which can be used to guide intervention design and to assess and understand context before an intervention is implemented.^16^ The generic protocol outlines three main activities: review of policy documents; readiness assessments; and qualitative research involving relevant stakeholders.^16^ The document review and readiness assessment are conducted to map existing guidelines, policies, protocols and birth-related care organizations. The qualitative research helps understand critical drivers of caesarean sections. Such research, which has been conducted in Burkina Faso,^17^ Romania^18^ and Thailand,^19^ helps identify existing challenges and opportunities related to reducing caesarean sections and understanding the needs, preferences and priorities of key stakeholders.

Underpinning the formative research protocol are theoretical frameworks of behaviour change.^16^ Such frameworks provide structured approaches to: understanding factors that influence behaviour; developing effective and appropriate interventions; and evaluating implementation.^12^^,^^20^ Many such behaviour change frameworks are available, such as theoretical domains framework and the capability, opportunity, and motivation of behaviour model.^20^^,^^21^ We recommend that a behaviour change framework is used during intervention design. The framework can identify the most appropriate interventions for a certain context, and can help articulate how and why intervention and implementation strategies may change behaviour.^12^

## Step 2: intervention design

The next step after conducting formative research is to design and then implement interventions. Deciding the target population for an intervention depends on available financing, but also on the results of formative research. For example, in settings where addressing the preferences of health workers has been identified as the most important challenge to optimizing caesarean use, interventions should be targeted at health workers. In settings where women’s fears and societal norms around birth are important factors, interventions should target women and families. Often however, core, interrelated issues are identified among all stakeholders, needing multifaceted interventions that address different groups.

### Identifying intervention components

A challenge with implementing multicomponent interventions is knowing which intervention components contributed to the intervention’s success or failure and how these might be related. For example, if an intervention included a review of caesarean section practices and provision of feedback to clinicians, as well as health education for women, it would be hard to know whether the observed effects are due to one component, both or neither.

**Fig. 1:**
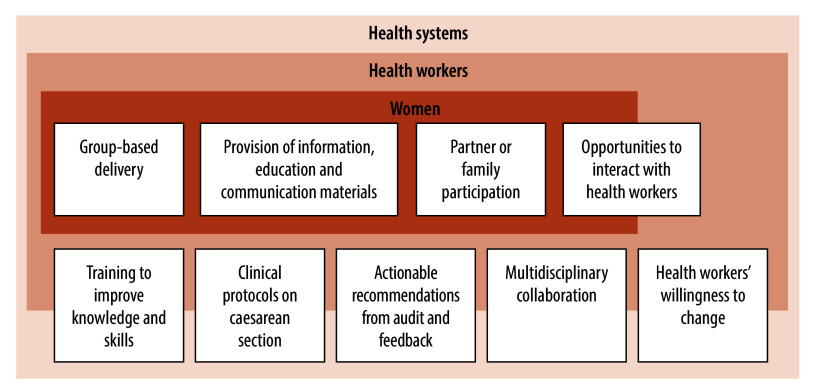
Key components of interventions that influence their success in optimizing the use of caesarean births

Qualitative comparative analysis is a recently developed research method that evaluates how different intervention components lead to specific outcomes such as intervention success or failure.^22^ We conducted two such analyses to evaluate interventions designed to optimize caesarean use.^14^^,^^15^ Using the analyses, we assessed which combinations of intervention components most commonly appeared in successful interventions. Fig. 1 shows the key components of successful interventions that emerged from our analyses, mapped according to the intervention target (women and health workers). In the following sections, we discuss how to combine these components to maximize the successful implementation of interventions.^14^^,^^15^

### Women

Interventions aimed at optimizing caesarean use that target women include childbirth training workshops, relaxation training and psychoeducation,^10^ all conducted during pregnancy. During these interventions, information is shared with women on: different birth methods; the stages of labour and vaginal birth; preparation for labour, such as addressing the fear of childbirth; managing labour pain; and encouraging the partner’s involvement.^10^^,^^23^^,^^24^ Our analysis suggests that when designing educational interventions targeting women, four key intervention components should be included to improve likelihood of success: group-based intervention delivery; provision of information, education and communication materials about childbirth to women; partner or family involvement; and opportunities to interact with health workers.^15^ Detailed definitions of each intervention component are presented in Table 1.

**Table 1:** Key intervention components when targeting women and health workers

Key intervention component	Description
**Educational interventions targeting pregnant women**	
Group-based delivery	Delivers education or support sessions facilitated in a group setting, allowing women to engage with peers, share experiences and learn collaboratively. Sessions often include information about different birth methods, the birth process, mental health and coping strategies, pain relief methods and partners’ roles in birth
Provision of information, education and communication materials about childbirth to women	Distributes evidence-based materials (such as leaflets, posters, videos, or digital content) designed to educate women about pregnancy and childbirth, supporting informed decision-making and preparation for birth
Partner or family involvement	Provides opportunities for partners or family members to be actively involved in the intervention and/or maternity care, including attending education sessions or consultations with health workers
Opportunities to interact with health workers	Implements one-on-one counselling that provides women with direct access to health workers, enabling them to ask questions, receive personalized guidance and build trust in the care process
**Interventions targeting health workers**	
Training to improve health worker knowledge and skills for labour management	Implements theory-based or practical education sessions aimed at improving health workers’ clinical knowledge and practical skills in evidence-based labour and delivery management
Clinical protocols for caesarean section (active dissemination of indications for caesarean births via protocols, guidance or algorithms)	Actively disseminates clear, standardized clinical protocols, decision-making tools, reminder systems, or information, education and communication materials that outline appropriate indications for caesarean births, promoting consistency and adherence to evidence-based practice
Actionable recommendations from audit and feedback	Implements structured processes where, at each audit and feedback cycle, health workers receive regular data-driven feedback on their clinical practices, paired with clear, practical recommendations for improvement to support accountability and quality enhancement
Multidisciplinary collaboration (between obstetricians, doctors, midwives and nurses)	Involves different cadres of health workers in caring for women
Ensuring health workers’ willingness to change^a^	Ensures that health workers’ willingness to adapt to change and adhere to the intervention is addressed

^a^ Willingness to change was added in our qualitative comparative analysis, as it becomes an overarching factor across qualitative evidence syntheses^25^^,^^26^ and the discussion section of the trials reports,^27^^,^^28^ where both health workers and trialists mentioned that the underlying factor of success lies on health workers’ beliefs about caesarean births and vaginal birth, as well as whether health workers are willing to step out of their comfort zone to change.

Delivering education in a group-based setting aligns with social support theory,^29^ providing women with peer and emotional support, developing confidence through shared learning, reducing feelings of isolation and uncertainty, enabling sharing of experiences, and improving health behaviours.^30^^,^^31^ Women also valued learning more about childbirth and expanding their understanding of the benefits and risks associated with both vaginal and caesarean birth.^32^ Women expressed that receiving information, education and communication materials about the mode of birth educated them about risks that they were not at all aware of previously and provided confidence and power in the birthing process.^32^ The materials also provide content to inform meaningful discussion with health workers and justify existing preferences on a certain mode of birth.^32^ Partner or family involvement is highly influential for emotional and instrumental support, facilitates shared decision-making and supports adherence to beneficial suggestions.^32^^,^^33^ Finally, women’s interactions with health workers are also critical as they provide women with personalized guidance, birth planning, support in decision-making and reassurance in professional presence; however, interactions can also be complex, involving power, trust and risk.^34^ While effective communication between women and health workers can improve shared decision-making, women may either trust providers as reliable sources of information or be sceptical of their influence.^34^ This underscores the need for health workers to be mindful of context and encourage meaningful conversations with women.^34^^,^^35^

**Fig. 2:**
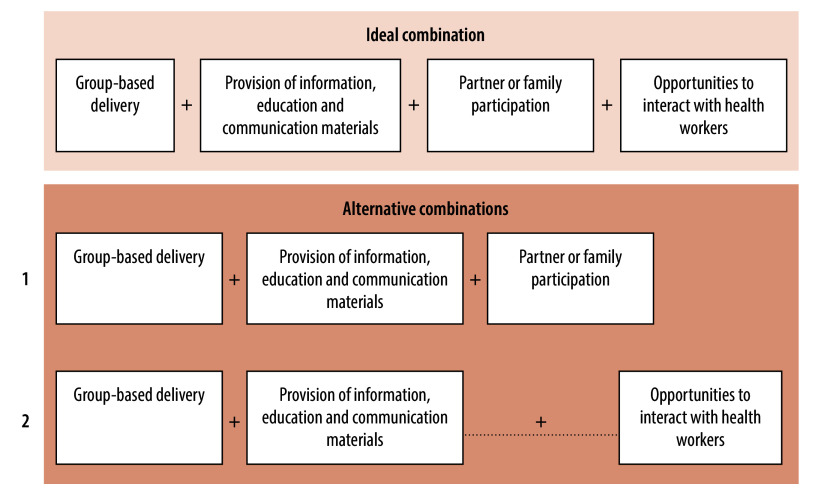
Combinations of intervention components targeting women and families that lead to the successful implementation of interventions

Our qualitative comparative analyses also suggested that interventions targeting women are most effective in reducing caesarean use when all four key components are implemented together (Fig. 2; ideal combination). We found that the absence of two or more of the four components in an intervention result in unsuccessful interventions (that is, no change in caesarean birth rate or an increase in caesarean birth rate). When one intervention component is missing, our analyses show that only two alternative combinations of intervention components influence success.^15^ In the first alternative combination, the presence of group-based intervention delivery, provision of information, education and communication materials to women, and partner or family involvement, can prompt a successful outcome (Fig. 2; alternative combination 1). However, in the second combination, when partner or family involvement is absent from the intervention, an opportunity to interact with health workers is needed for successful implementation (Fig. 2; alternative combination 2).

Initially, we hypothesized that engagement strategies (governing the intensity, frequency, technique, recruitment and incentives of an intervention) would be critical to intervention success. However, our analysis suggests that the consistent presence of support for women was a more important driver of intervention success. We found that while all intervention components were delivered during pregnancy, the timing (for example, second or third trimester) and mode of delivery (for example, community or health facility) varied across interventions. What proved most important was not when or how these components were delivered, but whether they were delivered at all. For example, partner or family involvement in the intervention was introduced at different times during pregnancy and through different approaches (e.g. partner involved in education sessions, partner involved in consultation only, or both), but it was the partner’s inclusion alone that was consistently associated with intervention success. Our findings suggest that simply integrating supportive elements may be more important than standardizing approaches on when and how interventions are delivered. Standardized approaches may not work as acceptability varies across health systems and cultural contexts, making formative research essential to ensure interventions are locally relevant and effective. This reflects the principle in the United Kingdom Medical Research Council framework on evaluating complex interventions:^36^ interventions can retain core functions, while allowing flexibility in intervention delivery, to adapt to context while safeguarding fidelity to the intervention’s purpose.

### Health workers

Among interventions aimed at optimizing caesarean use, those targeting health workers are most successful when they include the following five components: training to improve health worker knowledge and skills about labour management; clinical protocols for caesarean sections (active dissemination of indications for caesarean births via protocols, guidance or algorithms); actionable recommendations from audit and feedback; multidisciplinary collaboration (between obstetricians, other doctors, midwives and nurses); and ensuring health workers’ willingness to change.^14^ Descriptions of these intervention components are presented in Table 1.

Training, clinical protocols, audit and feedback, and multidisciplinary collaboration have all been widely implemented approaches to monitor caesarean rates.^10^ However, willingness to change among health workers is a comparatively new concept.^14^ Their willingness is often influenced by concerns about potential income loss, fear of blame for poor outcomes, professional status, or conflicting values.^25^ Because willingness to change influences individual attitudes and behaviour, it is critical for intervention success. Thus, willingness to change should be assessed through formative research before intervention implementation.

**Fig. 3:**
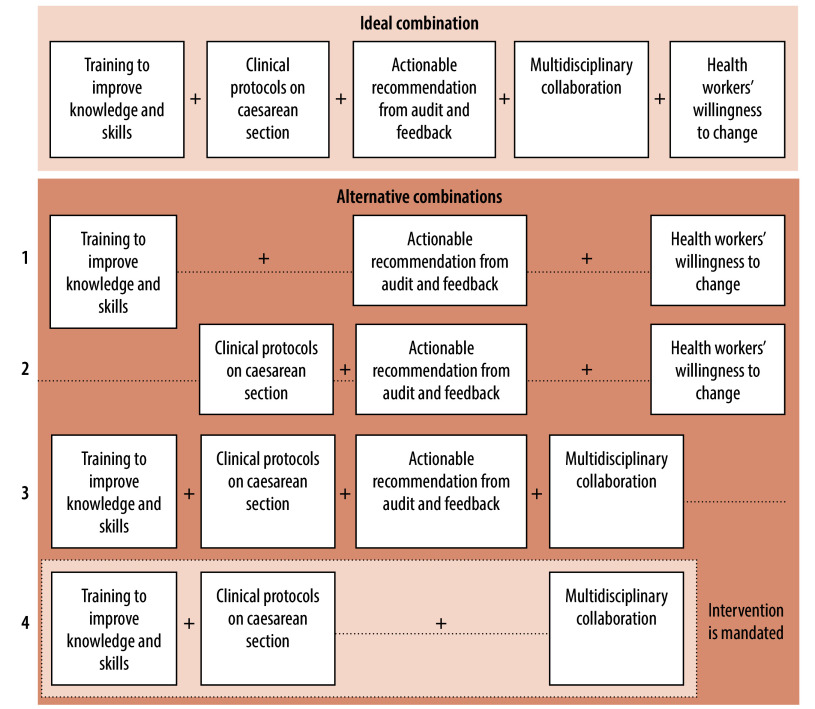
Combinations of intervention components targeting health workers that lead to the successful implementation of interventions

Our analysis shows that interventions targeting health workers are successful when all five intervention components are implemented together (Fig. 3; ideal combination). Our analysis also suggests that health workers’ willingness to change is a critical enabling factor. If any of the other four components are absent, willingness to change must be present for successful implementation (Fig. 3; combination 1 and 2). If willingness to change is absent, all four remaining components must be implemented to offset its absence (Fig. 3; combination 3). One way of mitigating health workers’ fears about blame and litigation may be multidisciplinary collaboration, in particular, collaborative decision-making and collegial support.^25^ Successful implementation may need a mandatory approach (for example, making it compulsory for health workers to adhere to interventions) if health workers are not willing to change and all the other components are not present (Fig. 3; combination 4).

## Step 3: engagement

The successful implementation of an intervention aimed at optimizing caesarean use also depends on how it is introduced and integrated into the routine functioning of health systems to achieve long-term success and sustainability.^37^ Here we outline several strategies that may help interventions aimed at optimizing caesarean sections use to be implemented, based on the United Kingdom National Institute of Health Research and Medical Research Council’s framework for complex interventions (hereafter referred to as the complex interventions framework).^36^

The complex interventions framework recommends iterative development of implementation strategies before scale-up.^36^ These include small-scale pilots, meaningful stakeholder engagement and robust process evaluations.

### Small-scale pilots

We suggest starting the implementation of interventions with small-scale pilots, for example, in a single health facility. Small-scale initiation, followed by an incremental approach, allows best practices and challenges to be identified and can inform effective scale-up.^36^^,^^38^ Starting small also allows risk mitigation and ensures that interventions are adaptable and sustainable ahead of larger-scale implementation.

### Meaningful stakeholder engagement

Meaningful stakeholder engagement should be integrated throughout an intervention’s lifecycle (particularly when stakeholders are women, partners or family health workers and policy-makers). The complex intervention framework highlights the importance of engaging stakeholders at every stage of intervention, as it is a core process to facilitate contextual fit, feasibility, adoption and sense of ownership.^36^^,^^39^^,^^40^ Importantly, stakeholder engagement fosters clear governance and leadership coordination and ensures interventions are aligned across sectors and integrated into routine care.^40^

### Robust process evaluations

Robust process evaluations show what works, why it works and can identify which intervention components contribute to success. Findings will inform improvements, scale-up and sustainability. The complex interventions framework emphasizes the importance of assessing process outcomes (fidelity, dose, reach, adaptation, acceptability and unintended consequences) via process evaluations.^38^ Evaluations should go beyond effectiveness outcome (that is, caesarean rates) to assessing how interventions are delivered and received. For example, it is essential to assess whether women received education sessions as intended, whether health workers consistently received recommendations from audit and feedback, and how the intervention was adapted over time.

These strategies are mutually reinforcing and provide a structured, theory-informed pathway to intervention implementation. We recommend the intentional use of established implementation frameworks to guide both the implementation and evaluation of interventions aimed at reducing unnecessary caesarean births.

## Providing high-quality care

Providing high-quality care is critical to optimizing caesarean use. The following can all help in this respect:^7^^,^^41^^–^^43^ access to safe and timely emergency obstetric care; respectful maternity care and continuous and emotional support for women; promotion of vaginal birth (when possible); implementation of evidence-based practices; avoidance of unnecessary interventions; and ensuring health workers are skilled and trained in physiological birth and can effectively manage labour complications. Creating a supportive and respectful birthing environment where women feel empowered to participate in decision-making, are supported emotionally throughout childbirth with labour companions and are treated respectfully through effective communication with health workers, may also help optimize use of caesarean sections.^41^^,^^42^ Therefore, ensuring that women receive high-quality, supportive, evidence-based care throughout pregnancy and childbirth goes hand-in-hand with efforts to reduce unnecessary caesarean sections.

## Conclusion

Every pregnancy and birth is unique, and while caesarean sections are medically needed in certain situations, promoting vaginal birth where possible can lead to significant benefits for both women and babies. Effective interventions aimed at optimizing caesarean use are likely to be multifaceted. We outline ways in which the rising trend for caesarean sections might be addressed, most notably through a three-step pathway for implementing interventions that aim to optimize caesarean use. Formative research before implementing interventions can help to identify and address context-specific challenges. Intervention components that target both women and health workers are important; using behaviour change and implementation science frameworks, and involving key stakeholders throughout intervention design, implementation and evaluation are all key to success. During implementation, process evaluations can help to understand what does or does not work, and why. Additionally, improving the quality of childbirth care is essential for optimal use of caesarean sections, high-quality, supportive care throughout labour and birth has the potential to empower women and promote positive childbirth experiences. In the absence of these considerations, women and health workers may opt for caesarean birth as a perceived safer, less painful, or more predictable alternative to vaginal birth.
